# Soil phosphorus stocks could prolong global reserves and improve water quality

**DOI:** 10.1038/s43016-024-01086-8

**Published:** 2025-01-02

**Authors:** R. W. McDowell, P. M. Haygarth

**Affiliations:** 1 Faculty of Agriculture and Life Sciences, Lincoln University, Christchurch, New Zealand; 2 Environmental Sciences, AgResearch, Christchurch, New Zealand; 3 Lancaster Environment Centre, Lancaster University, Lancaster, UK

**Keywords:** Element cycles, Environmental studies, Agriculture

## Abstract

Combining existing databases, we estimated global phosphorus stocks in croplands and grasslands that are not readily available to plants as 32–41% of the 2020 estimated geologic phosphorus reserves, representing 146–186 years of the 2020 mass of phosphorus fertilizer applied annually. Especially if accessed by more efficient crops, this stock could reduce the need for additional fertilizer, improve water quality and contribute to all-round phosphorus sustainability.

## Abstract (web summary)

Combining existing databases, this study estimates global phosphorus stocks in croplands and grasslands that are not readily available to plants. The reserves represent 146–186 years of the 2020 mass of phosphorus fertilizer applied annually.

## Main

Human existence depends on phosphorus fertilizers to produce food^1^. These fertilizers arise from geologic phosphorus supplies extracted from relatively few geopolitical locations, and require processing, transportation and distribution, before application to land. In many countries, large applications of phosphorus have been applied as ‘insurance’ against poor phosphorus utilization caused by factors such as strong soil phosphorus sorption and slow crop uptake^2^. This has resulted in soils that may contain readily plant available phosphorus at or above the optimum level for crop growth, but also considerable stocks of phosphorus that are not readily available^3^.

Existing studies have already shown that, with improvements in crop acquisition and uptake through a combination of conventional breeding and genetic engineering, these non-readily available stocks could sustain crop production for many years with much lower phosphorus applications^4^. Furthermore, because the risk of losing dissolved and particulate forms of phosphorus via runoff and leaching from topsoil increases with soil phosphorus concentrations, reducing topsoil concentrations and fertilizer applications will help improve surface water quality^5^. Nevertheless, exact estimates are lacking.

**Fig. 1:**
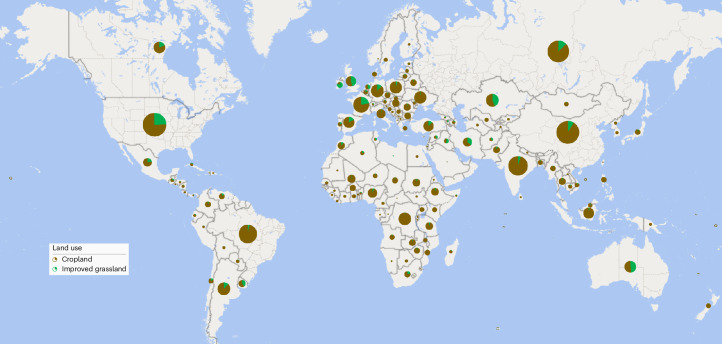
Map of non-readily plant available phosphorus. Map showing the relative stock of non-readily plant available phosphorus (3.510 Gt) in the top 20–30 cm of soil by land use. Data are the mean calculated values from Ringeval et al.^6^ and McDowell et al.^7^. The base map used data sourced from OpenStreetMap contributors available under an Open Database License (https://www.openstreetmap.org/copyright).

Here, we hypothesize that, globally, there is sufficient phosphorus in soils that if used efficiently could be exported in crop produce, rather than to surface waters, and significantly offset the need to apply new fertilizer. To test this hypothesis, we calculated the stock of total, readily and non-readily plant available phosphorus in the top 20–30 cm of cropland and improved grassland soils from two new databases^6,7^. We estimated that globally, for croplands and improved grasslands, the total phosphorus stock—as the range between the two databases—was 3.32–4.26 Gt, of which 3.00–3.83 Gt was non-readily available. On a continent basis, the greatest mean non-readily plant available stock was present in Europe (Table 1), caused by phosphorus-enriched cropland. Across countries, stocks reflected land areas, but the greatest stocks for cropland and improved grassland are dominated by European countries, Canada, the USA and China (Fig. 1), reflecting a long legacy of phosphorus fertilizer application^8^.

**Table 1:** Mean continental stocks in Gt (in the top 20–30 cm depth) of plant available and non-readily plant available soil phosphorus in cropland and improved grassland

Continent	Cropland		Improved grassland		Other land uses
	Plant available	Non-readily plant available	Plant available	Non-readily plant available	Total
Africa	0.037	0.546	0.002	0.027	0.837
Asia	0.089	0.844	0.007	0.119	0.901
Europe	0.070	0.934	0.007	0.147	1.227
North America	0.024	0.334	0.005	0.098	1.174
Oceania	0.083	0.055	0.003	0.035	0.258
South America	0.027	0.335	0.002	0.034	0.454
Total	0.254	3.049	0.024	0.461	4.851

Data were sourced from the 0–20 cm depth from McDowell et al.^7^ and Ringeval et al.^6^. The mean year of these databases is 2015.

We also calculated phosphorus stocks in forest and rangeland or non-productive land (using the database of McDowell et al.^7^). Inclusion of this phosphorus yielded a total global stock of 8.64 Gt. We did not consider the conversion and utilization of stocks in non-productive areas for crops or improved grassland because non-productive areas are probably limited by other factors such as climate^9^. In addition, conversion would also increase carbon emissions^10^ and nutrient and sediment losses^11^. However, the global stock was useful to compare against other published stocks (8–200 Gt (refs. ^12,13^)). Although at the low end of this range, our calculation is robust given our focus on topsoil that is accessible to crops as opposed to deeper soil layers (included in other studies; for example, refs. ^14–16^), and our large and spatially distributed databases (see Methods for further detail).

In 2020, the global estimated stock of phosphate rock reserves was 71 Gt, which equated to 9.30 Gt of phosphorus after accounting for the P_2_O_5_ concentration in phosphate rock (30%) and conversion of P_2_O_5_ into phosphorus^17^. Our estimate of the stock for cropland and improved grassland (Table 1) represents 32–41% of the 2020 estimated geologic phosphorus reserves. This is a sizeable and unused resource^18^ that if accessed by agricultural plants is equivalent to 146–186 years of the 2020 global mass of phosphorus fertilizer applied annually (0.0205 Gt).

This estimate is likely to be conservative with additional work showing that savings could also be made by a combination of lowering application rates to meet Olsen phosphorus concentrations considered optimal for plant growth, increasing stocks where currently deficient to support optimal growth^3^, utilizing more efficient cultivars^19^, adding additives (for example, phosphorus-solubilizing microbes)^20^, and adjusting where crops are grown for better phosphorus-use efficiency, yield and adaptation to climate change, especially in areas of Africa where yields can be poor^21,22^. Consideration must also be given to those phosphorus-rich soils for which the liberation and inefficient crop uptake of phosphorus could enhance phosphorus losses to water and the potential for eutrophication^23^. This will require farmers and extension services to carefully match crop requirements and uptake to local climates and soil types, along with regulatory and supportive policies in areas where change is necessary. There is undoubtedly an opportunity to utilize indigenous and applied soil phosphorus more efficiently and tighten local and global phosphorus cycles.

## Methods

We compiled data from two published datasets of soil phosphorus concentrations in topsoil. The dataset of Ringeval et al.^6^ reported total phosphorus and constituent inorganic and organic phosphorus fractions to 30 cm depth. The dataset of McDowell et al.^7^ reported Olsen plant available phosphorus to 20 cm. Both datasets identified cropland as per Monfreda et al.^24^ and Grogan et al.^25^ at the same resolution (0.5° × 0.5°). Additional work (below) was required to generate a consistent estimate of phosphorus stocks in cropland and improved grassland for both datasets, and to convert plant available soil phosphorus into total phosphorus for the McDowell et al.^7^ dataset.

We assessed potentially usable phosphorus as the difference between total phosphorus and Olsen phosphorus (in the McDowell et al.^7^ dataset) and between total phosphorus and the first readily available pool of phosphorus in the Ringeval et al.^6^ dataset. Because the McDowell et al.^7^ dataset also assessed other land uses, we used those data to check our calculations by calculating a global phosphorus stock (excluding polar regions) and compared these to other published estimates.

### Land uses

We combined a range of geographic databases to identify unique land uses and estimate phosphorus stocks. Although we calculate a global total phosphorus stock, we focus on cropland and improved grassland because considering the expansion of intensive agriculture onto other land may lead to deterioration of water, air or soil quality.

Due to the different spatial resolutions and degrees of accuracy, spatial data were applied in the following order: (1) rangeland was classified according to the Food and Agriculture Organization’s (FAO) rangeland class for livestock^26^; (2) forestland, defined as evergreen or deciduous broadleaf-tree-covered areas, was identified on the basis of data from the European Space Agency^27^; (3) improved grassland was classified based on the European Space Agency grassland class^27^ if indicated as cropland within the NASA 2010 World Cropland database^28^, which includes improved grasslands but not rangelands; (4) all other crops were distributed within the NASA cropland class according to the spatial distributions described by Monfreda et al.^24^ and Grogan et al.^25^; and (5) non-productive land was categorized as all other land. The FAO and European Space Agency data were mapped at a resolution of at least 1 km^2^. NASA data were mapped at a resolution of 30 m^2^. The data of Monfreda et al.^24^ had a resolution of 100 km^2^, which improved to about 9 km^2^ with the use of data from Grogan et al.^25^. Land parcels were ascribed to the most likely land cover type within the most spatially refined class.

### Total phosphorus stock from plant available phosphorus

We calculated the stock of soil Olsen phosphorus by multiplying a map of topsoil (0–20 cm) Olsen phosphorus concentrations at 1 km^2^ resolution^7^, created from a verified and filtered database of ~32,000 observations, with bulk density data^29^ for the top 20 cm of soil. We chose these data because Olsen phosphorus is the most widespread test of plant available phosphorus, and the map was the most spatially well refined and representative outputs of topsoil phosphorus concentrations available. We converted these data to total phosphorus in recognition that Olsen phosphorus may not be the best test of plant available phosphorus nor represent the total amount of phosphorus available to plants^30^.

To estimate the total topsoil phosphorus stock, we multiplied the Olsen phosphorus concentrations by a ratio of soil Olsen to total phosphorus concentrations. Studies with data for the same samples on soil Olsen and total phosphorus concentrations are rare. However, we scoured the literature to find 19 studies with such data for topsoils (0–20 cm; Supplementary Table 1). To maximize spatial representativeness, we only considered those with data for multiple soil types, although coordinates were seldom available to confirm this. While we were able to extract raw data from half the studies or databases, the remainder only reported linear regressions between Olsen and total phosphorus significant at least the *P* < 0.05 level. We therefore extracted the slope of those regressions and generated significant (*P* < 0.05) linear regressions for the extracted data. We combined all data into one database of 1,183 observations covering 13 jurisdictions and >50 different soil types, including well-leached tropical soils. Inserting a term in our model to test if the slopes of each regression were different showed no significant differences except for the data from Guatemala which had a lower slope than that from Central Europe. However, we removed the Guatemalan data on the basis that they were derived from low-productivity (not improved) grassland. Although we could not test the slopes for extracted data against the reported linear regressions, most of the regressions were derived from extracted data (*n* = 659). We therefore pooled the outputs of both sets of regressions and generated a weighted-mean slope of 0.039. We set the intercept at zero given that the intercept in the regression of the extracted data was not significantly different from zero.

There is potential for variation when converting between plant available phosphorus and total phosphorus, especially at low concentrations of plant available phosphorus^31^. The data calculated from McDowell et al.^7^ and other data support the use of a conversion ratio of ~4% Olsen phosphorus to total phosphorus^32,33^. Note that the percentages for cropland (7%) and improved grassland (5%) in Table 1 are slightly larger due to the combining of the two databases. One study in Spain estimated that Olsen phosphorus could be 25% of total phosphorus especially at low concentrations^34^. However, we considered this localized and unrepresentative of global relationships between Olsen and total phosphorus.

To check that our estimate of total soil phosphorus stock is sensible we calculated the phosphorus stock to 20 cm depth assuming a total phosphorus concentration of 250 mg kg^−1^ (estimated as an approximate cut-off between anthropogenic and non-anthropogenic-influenced land^35^) and a bulk density of 1.2 g cm^−3^. Total land area is 148,940,000 km^2^, which equates to a mass of 2.97 × 10^16^ kg of soil. Using our total phosphorus concentration of 250 mg kg^−1^, this equates to an estimated stock of 8.9 Gt (8,936,400 kt), which is only 9% greater than our estimated stock. This seems sensible given most global land is either forest, rangeland or non-productive, which would have low total phosphorus concentrations. Other estimates of the soil phosphorus stock, excluding any fluxes associated with biogeochemical processes (for example, mineralization and leaching) are 8 Gt (ref. ^12^), 16 Gt (ref. ^36^), 39 Gt (refs. ^14,37^), 40 Gt (ref. ^15^) and 200 Gt (ref. ^13^). However, these are not comparable to our estimated stock of 8.2 Gt for three main reasons. First, these estimates calculate stock to depths of 50 cm or more. Second, stocks at deeper depths would be inflated by greater soil bulk density. Over the 15 profiles considered by the most recent studies^14,37^, bulk density was on average 2.4 g cm^−3^, much greater than the mean bulk density of topsoil, commonly around 1.2 g cm^−3^ for cropland and improved grassland^29^. Third, all are global biogeochemical studies that model soil phosphorus stocks for different soil orders, largely from the weathering rates of phosphorus from parent material. Weathering rates are derived from the mean of a few (*n* = 14) studies of 29 chronosequences. The chronosequences are not well distributed around the world, are largely restricted to the USA and New Zealand, and are often not on agricultural land where the stock of phosphorus is strongly controlled by crop offtakes and phosphorus inputs as fertilizers and manure^38^.

Using the same calculation logic for the most recent estimate above (40 Gt), but assuming a more agronomically realistic bulk density of 1.2 g cm^−3^, rather than 2.4 g cm^−3^, we estimate the mean global total phosphorus concentration to be ~1,113 mg kg^−1^. Although this calculated concentration would be feasible in cropland and some improved grasslands (for example, those used for high-intensity dairy farming^39^), it is well above that expected for forest, rangeland (Olsen phosphorus commonly is <5 mg kg^−1^ in native forests and rangeland with total phosphorus concentrations of 100–250 mg kg^−1^ (refs. ^40,41^)), and non-productive land (for example, Olsen phosphorus in the Sahara Desert is ~2 mg kg^−1^, equivalent to a total phosphorus of 50 mg kg^−1^ (ref. ^42^)). Some dilution of this total phosphorus concentration could be accounted for by considering that most of the stock will be concentrated in shallower soil horizons, where phosphorus inputs by fertilizer and manure occur. However, we contest that our data for global total phosphorus stock, despite being calculated using a ratio of Olsen to total phosphorus, is more robust given that the underlying Olsen phosphorus data are far more spatially representative of soil types and land management practices than the biogeochemical modelling approaches listed above.

### Reporting summary

Further information on research design is available in the Nature Portfolio Reporting Summary linked to this article.

## Supplementary information

Supplementary Information **Supplementary Table 1. Slope, coefficient of determination (*r***^***2***^**) and the number of samples (N) used in linear regression relationships between soil Olsen and total phosphorus concentrations**. Data for regression parameters were either sourced from each report or generated from the raw data (*), where available. Note that data from Guatemala were identified as an outlier and not included in the final regression relationship.

Reporting Summary

## Data Availability

All data from McDowell et al.^7^ are available via Figshare at 10.6084/m9.figshare.14241854. All data from the Ringeval et al.^6^ database are available via Figshare at 10.57745/XZTW7Z. The summary data for the relationship between Olsen and total phosphorus and the estimate of soil phosphorus stocks by land use are available via Figshare at 10.6084/m9.figshare.22137506.v1 (ref. ^43^).

## Code availability

All GIS code used to isolate land-use stocks from McDowell et al.^7^ and Ringeval et al.^6^ is available via Figshare at 10.6084/m9.figshare.14241854 and 10.57745/XZTW7Z, respectively. The spreadsheet used to generate the relationship between Olsen and total phosphorus and the estimate of soil phosphorus stocks by land use is available via Figshare at 10.6084/m9.figshare.22137506.v1 (ref. ^43^).
