# Supplementary material for: Soil phosphorus stocks could prolong global reserves and improve water quality
Source: Nat Food. 2025 Jan 2;6(1):31–5. doi: 10.1038/s43016-024-01086-8 (PMC11772246; doi:10.1038/s43016-024-01086-8)
Supplement: Supplementary file 1 — Supplementary Table 1. Slope, coefficient of determination (r2) and the number of samples (N) used in linear regression relationships between soil Olsen and total phosphorus concentrations. Data for regression parameters were either sourced from each report or generated from the raw data (*), where available. Note that data from Guatemala were identified as an outlier and not included in the final regression relationship. [file 43016_2024_1086_MOESM1_ESM.pdf]

nature food

Supplementary information https://doi.org/10.1038/s43016-024-01086-8

Soil phosphorus stocks could prolong global reserves and improve water quality

In the format provided by the authors and unedited

Supplementary Information

Soil phosphorus stocks could prolong global reserves and improve water quality

R.W. McDowell[1,2], P.M. Haygarth[3]

Table S1. Slope, coefficient of determination (r^2) and the number of samples (N) used in linear regression relationships between soil Olsen and total phosphorus concentrations. Data for regression parameters were either sourced from each report or generated from the raw data (*), where available. Note that data from Guatemala were identified as an outlier and not included in the final regression relationship.

Study	Slope (Olsen P / total P)	r^2	N	Jurisdiction	Reference
1	0.057	0.44	50	Austria/Germany	1
2	0.023	0.26	113	China	2
3	0.044	0.69	52	China	3
4	0.041	0.47	52	China	3
5	0.047	0.53	52	China	3
6	0.089	0.53	108	China	4
7	0.064	0.66	24	US	5
8	0.039	0.32	32	US	5
9	0.051	0.90	9	US	5
10*	0.041	0.56	263	US	6
11*	0.062	0.90	8	US	7
12*	0.028	0.57	168	New Zealand	8
13*	0.006	0.15	54	China	9
14*	0.021	0.63	14	China	10
15*	0.039	0.52	44	UK/India/Egypt	11-13
16*	0.043	0.44	50	Central Europe	14
17*	0.017	0.53	35	Guatemala	15
18*	0.043	0.67	24	UK/Italy/Austria/Hungary	16
19*	0.037	0.62	32	UK	17
Weighted mean, sum	0.039	0.52	1182		

References

1 Wuenscher, R., Unterfrauner, H., Peticzka, R. & Zehetner, F. A comparison of 14 soil phosphorus extraction methods applied to 50 agricultural soils from Central Europe. *Plant, Soil and Environment* **61**, 86-96 (2015).

2 Zhang, S., Huffman, T., Zhang, X., Liu, W. & Liu, Z. Spatial distribution of soil nutrient at depth in black soil of Northeast China: a case study of soil available phosphorus and total phosphorus. *J. Soils Sed.* **14**, 1775-1789 (2014).

3 Wu, Q. *et al.* Determining the optimum range of soil Olsen P for high P use efficiency, crop yield, and soil fertility in three typical cropland soils. *Pedosphere* **30**, 832-843 (2020).

4 Wang, R. *et al.* Phosphorus Accumulation and Sorption in Calcareous Soil under Long-Term Fertilization. *PLoS ONE* **10**, e0135160 (2015).

5 Allen, B. L. & Mallarino, A. P. Relationships between extractable soil phosphorus and phosphorus saturation after long-term fertilizer or manure spplication. *Soil Sci. Soc. Am. J.* **70**, 454-463 (2006).

6 National Cooperative Soil Survey. National Cooperative Soil Survey Characterization Database. (2020).

7 Ohno, T., Griffin, T. S., Liebman, M. & Porter, G. A. Chemical characterization of soil phosphorus and organic matter in different cropping systems in Maine, U.S.A. *Agric., Ecosyst. Environ.* **105**, 625-634 (2005).

8 Taylor, M. D., Kim, N. D., Hill, R. B. & Chapman, R. A review of soil quality indicators and five key issues after 12 yr soil quality monitoring in the Waikato region. *Soil Use. Manage.* **26**, 212-224 (2010).

9 Chinese Ecosystem Research Network 1998-2010. *Soil nutrient data of major farmland ecosystems in China (1990-2006)*, <http://soil.geodata.cn/data/datadetails.html?dataguid=118007709206684&docId=31> (2019).

10 Chen, X. *et al.* Long-term excessive phosphorus fertilization alters soil phosphorus fractions in the acidic soil of pomelo orchards. *Soil Tillage Res.* **215**, 105214 (2022).

11 Turner, B. L. & Haygarth, P. M. Phosphorus forms and concentrations in leachate under four grassland soil types. *Soil Sci. Soc. Am. J.* **64**, 1090-1099 (2000).

12 Ali, M. M. E.-S. Efficiency of Elemental Sulfur and Phosphorus Fertilizer in Enhancing Soybean (Glycine max L.) Growth and Yield in a Clayey Soil. *Egyptian Journal of Soil Science* **58**, 233-244 (2018).

13 Bhattacharyya, R. *et al.* Fertilization effects on yield sustainability and soil properties under irrigated wheat–soybean rotation of an Indian Himalayan upper valley. *Nutrient Cycling in Agroecosystems* **86**, 255-268 (2010).

14 Lair, G. J., Zehetner, F., Khan, Z. H. & Gerzabek, M. H. Phosphorus sorption-desorption in alluvial soils of a young weathering sequence at the Danube River. *Geoderma* **149**, 39-45 (2009).

15 Terry, R. E. *et al.* Quantitative phosphorus measurement: A field test procedure for archaeological site analysis at Piedras Negras, Guatemala. *Geoarchaeology* **15**, 151-166 (2000).

16 Withers, P. J. A. *et al.* An environmental soil test to estimate the intrinsic risk of sediment and phosphorus mobilization from European soils. *Soil Use. Manage.* **23**, 57-70 (2007).

17 Stutter, M. I. *et al.* Land use and soil factors affecting accumulation of phosphorus species in temperate soils. *Geoderma* **257-258**, 29-39 (2015).
