# Supplementary material for: Soil phosphorus stocks could prolong global reserves and improve water quality
Source: Nat Food. 2025 Jan 2;6(1):31–5. doi: 10.1038/s43016-024-01086-8 (PMC11772246; doi:10.1038/s43016-024-01086-8)
Supplement: Supplementary file 2 — Reporting Summary [file 43016_2024_1086_MOESM2_ESM.pdf]

nature portfolio

Corresponding author(s): RW McDowell

Last updated by author(s): Oct 31, 2024

Reporting Summary

Nature Portfolio wishes to improve the reproducibility of the work that we publish. This form provides structure for consistency and transparency in reporting. For further information on Nature Portfolio policies, see our Editorial Policies and the Editorial Policy Checklist.

Statistics

For all statistical analyses, confirm that the following items are present in the figure legend, table legend, main text, or Methods section.

n/a	Confirmed	
☐	☒	The exact sample size (*n*) for each experimental group/condition, given as a discrete number and unit of measurement
☒	☐	A statement on whether measurements were taken from distinct samples or whether the same sample was measured repeatedly
☒	☐	The statistical test(s) used AND whether they are one- or two-sided<br>*Only common tests should be described solely by name; describe more complex techniques in the Methods section.*
☒	☐	A description of all covariates tested
☒	☐	A description of any assumptions or corrections, such as tests of normality and adjustment for multiple comparisons
☐	☒	A full description of the statistical parameters including central tendency (e.g. means) or other basic estimates (e.g. regression coefficient) AND variation (e.g. standard deviation) or associated estimates of uncertainty (e.g. confidence intervals)
☐	☒	For null hypothesis testing, the test statistic (e.g. *F*, *t*, *r*) with confidence intervals, effect sizes, degrees of freedom and *P* value noted<br>*Give P values as exact values whenever suitable.*
☒	☐	For Bayesian analysis, information on the choice of priors and Markov chain Monte Carlo settings
☒	☐	For hierarchical and complex designs, identification of the appropriate level for tests and full reporting of outcomes
☒	☐	Estimates of effect sizes (e.g. Cohen's *d*, Pearson's *r*), indicating how they were calculated

Our web collection on statistics for biologists contains articles on many of the points above.

Software and code

Policy information about availability of computer code

Data collection	No software was used.
Data analysis	No bespoke code was used in our analysis beyond calculating the stock of soil phosphorus from recently published data.

For manuscripts utilizing custom algorithms or software that are central to the research but not yet described in published literature, software must be made available to editors and reviewers. We strongly encourage code deposition in a community repository (e.g. GitHub). See the Nature Portfolio guidelines for submitting code & software for further information.

Data

Policy information about availability of data

All manuscripts must include a data availability statement. This statement should provide the following information, where applicable:

- Accession codes, unique identifiers, or web links for publicly available datasets
- A description of any restrictions on data availability
- For clinical datasets or third party data, please ensure that the statement adheres to our policy

All data and GIS code used to isolate land use stocks from McDowell, et al. (2023) are available at: https://doi.org/10.6084/m9.figshare.14241854. The data and code for the Ringeval, et al. (2024) database are available at: https://doi.org/10.57745/XZTW7Z. The summary data and spreadsheet used to generate these estimates is available here: https://figshare.com/s/45bfcc1301b69c34409a.

Human research participants

Policy information about studies involving human research participants and Sex and Gender in Research.

Reporting on sex and gender	na
Population characteristics	na
Recruitment	na
Ethics oversight	na

Note that full information on the approval of the study protocol must also be provided in the manuscript.

Field-specific reporting

Please select the one below that is the best fit for your research. If you are not sure, read the appropriate sections before making your selection.

☐ Life sciences ☐ Behavioural & social sciences ☒ Ecological, evolutionary & environmental sciences

For a reference copy of the document with all sections, see nature.com/documents/nr-reporting-summary-flat.pdf

Ecological, evolutionary & environmental sciences study design

All studies must disclose on these points even when the disclosure is negative.

Study description	We combined the most up to date information regarding soil plant available and total phosphorus to calculate the stock of soil phosphorus in croplands and improved grasslands. We compared these stocks to phosphorus reserves.
Research sample	We used modelled projections of mean global topsoil (0-20 cm) plant available phosphorus concentrations (thereafter termed Olsen phosphorus) at 1-km2 resolution and equations to convert these concentrations to total soil phosphorus. We combined these point data with another database of modelled estimates of soil total phosphorus concentrations and converted the mean of the intersection of both databases to soil phosphorus stocks. We used these data comment on how much soil phosphorus could extend global phosphorus reserves.
Sampling strategy	We combined two new database for global Olsen and total phosphorus at 1-km2.
Data collection	*Describe the data collection procedure, including who recorded the data and how.*
Timing and spatial scale	Data for the combined global soil Olsen phosphorus had a mean collection year of 2014 but ranged from 2000 to 2020
Data exclusions	A filtering regime was used to affirm the quality of the two databases and a check made of the conversions between Olsen P and total P . Data from the McDowell et al. (2023) database were excluded for non cropland or improved grassland land uses.
Reproducibility	We checked the validity of conversions between Olsen phosphorus and total phosphorus for the McDowell et al. (2023) database with independent point-based data that had both forms measured.
Randomization	Not applicable as the data were bespoke to specific points.
Blinding	Blinding was not applicable to this study. We describe our filtering rules in detail to constrain the data and avoid bias as much as possible.

Did the study involve field work? ☐ Yes ☒ No

Reporting for specific materials, systems and methods

We require information from authors about some types of materials, experimental systems and methods used in many studies. Here, indicate whether each material, system or method listed is relevant to your study. If you are not sure if a list item applies to your research, read the appropriate section before selecting a response.

Materials & experimental systems

n/a	Involved in the study
☒	☐ Antibodies
☒	☐ Eukaryotic cell lines
☒	☐ Palaeontology and archaeology
☒	☐ Animals and other organisms
☒	☐ Clinical data
☒	☐ Dual use research of concern

Methods

n/a	Involved in the study
☒	☐ ChIP-seq
☒	☐ Flow cytometry
☒	☐ MRI-based neuroimaging
